# Oxidative Stress in Ischemia/Reperfusion Injuries following Acute Ischemic Stroke

**DOI:** 10.3390/biomedicines10030574

**Published:** 2022-03-01

**Authors:** Anamaria Jurcau, Adriana Ioana Ardelean

**Affiliations:** 1 Department of Psycho-Neurosciences and Rehabilitation, Faculty of Medicine and Pharmacy, University of Oradea, 410087 Oradea, Romania; 2 Department of Neurology, Clinical Municipal Hospital Oradea, Louis Pasteur Street nr 26, 410054 Oradea, Romania; 3 Department of Preclinical Sciences, Faculty of Medicine and Pharmacy, University of Oradea, Universitatii Street nr 1, 410087 Oradea, Romania; adriana_toadere@yahoo.com; 4 Department of Cardiology, Clinical Emergency County Hospital Oradea, Gh. Doja Street nr 65, 410169 Oradea, Romania

**Keywords:** ischemic stroke, reactive oxygen species, mitochondria, oxidative stress, antioxidants, nanoparticles, stem cells

## Abstract

Recanalization therapy is increasingly used in the treatment of acute ischemic stroke. However, in about one third of these patients, recanalization is followed by ischemia/reperfusion injuries, and clinically to worsening of the neurological status. Much research has focused on unraveling the involved mechanisms in order to prevent or efficiently treat these injuries. What we know so far is that oxidative stress and mitochondrial dysfunction are significantly involved in the pathogenesis of ischemia/reperfusion injury. However, despite promising results obtained in experimental research, clinical studies trying to interfere with the oxidative pathways have mostly failed. The current article discusses the main mechanisms leading to ischemia/reperfusion injuries, such as mitochondrial dysfunction, excitotoxicity, and oxidative stress, and reviews the clinical trials with antioxidant molecules highlighting recent developments and future strategies.

## 1. Introduction

Although the proper management of vascular risk factors and increasing use of prophylactic measures between the 1970s and early 2000s resulted in an annual 1–1.5% decrease in stroke incidence in high income countries [1], stroke is still the main cause of disability in adults and the second leading cause of death worldwide. Moreover, the increasing prevalence of diabetes mellitus [2] and obesity [3], together with aging of the population, will probably increase the incidence of stroke [4].

The treatment of ischemic stroke entered a new era in 1995, with the release of the results of the National Institute of Neurological Disorders and Stroke trial with recombinant tissue plasminogen activator (r-tPA) [5], which showed that reestablishing blood flow in the first 3 h after stroke onset is able to salvage much of the hypoperfused cerebral tissue and improve patient outcome. Subsequent trials refined the recanalization methods by extending the time window to 4.5 h in certain subsets of patients [6], using intra-arterial thrombolysis [7], ultrasound-enhanced thrombolysis [8], or various devices for mechanical clot extraction [9] within 24 h from stroke onset [10].

However, still only 2–20% of acute ischemic stroke patients are eligible for recanalization treatments [11]. In addition, successful recanalization rates vary around 46% for intravenous thrombolysis, 63% for intra-arterial thrombolysis, or 83% for mechanical thrombectomy, and recanalization does not always translate into efficient reperfusion of the tissue at risk, leading to neurological worsening of the patient through cerebral edema, hemorrhagic transformation, or ischemia/reperfusion injuries (I/R injuries) [12]. Oxidative stress and neuroinflammation have been shown to significantly contribute to these complications. Thus, understanding the mechanisms of I/R injuries and finding ways to prevent them would significantly improve the outcome of ischemic stroke patients [13].

In the following sections we will review the literature on the pathophysiology of these injuries, focusing on oxidative stress, sources of reactive oxygen species (ROS), and neurotoxic oxidative and neuroprotective antioxidative pathways in the central nervous system (CNS). The second part reviews the studies done so far with antioxidants in ischemic stroke and discusses promising novel antioxidant approaches. 

## 2. Oxidative Stress in the Pathophysiology of Ischemia/Reperfusion Injuries after Acute Ischemic Stroke

Oxidative stress is an imbalance between the rate of generation of ROS and the biological system’s ability to clear these highly reactive molecules [14]. The cerebral tissue is particularly sensitive to oxidative stress due to a series of features, such as [15,16,17,18]:

‑ It has the highest metabolic activity per unit weight compared to other organs;
‑ It has low levels of antioxidant enzymes, such as superoxide dismutase, catalase, glutathione peroxidase, heme oxygenase-1;
‑ Upon release, neurotransmitters contribute to cellular calcium overload and, through their metabolism, generate ROS;
‑ Brain cells have a higher membrane surface/cytoplasmic volume ratio, and the plasmalemma is rich in cholesterol, is arranged in lipid rafts, has polyunsaturated fatty acids, and is very susceptible to oxidative damage;
‑ The brain has lower levels of cytochrome c oxidase, leading to increased superoxide generation during adenosine triphosphate (ATP) generation;
‑ Iron, released from damaged cerebral tissue, can catalyze the generation of free radicals.

Restoration of blood supply to ischemic tissue, although necessary for restoration of aerobic metabolism, will also result in ROS production, which overwhelms the ability of cerebral tissue to neutralize these ROS and leads to increased oxidative stress. Research has shown that cerebral ischemia is accompanied by increased serum concentrations of markers of oxidative stress [19,20,21].

The main ROS are superoxide anions, (O_2_^−^), hydroxyl radicals (OH^−^), and hydrogen peroxide (H_2_O_2_) [22], stemming from the activity of mitochondria, cyclooxygenases, lipoxygenases, nitric oxide synthases (NOSs), NADPH oxidase (NOX), and xanthine oxidase [23]. Once generated, ROS interact with various biological molecules:

‑ ROS oxidize, degrade, or cleave proteins, leading to protein aggregation, modifications in ion channel activities, and enzyme inactivation [24].
‑ By attacking the carbon–carbon bonds of polyunsaturated fatty acids, ROS initiate lipid peroxidation, a self-propagating chain of events leading to the generation of unstable lipid radicals which further react with oxygen to form lipid peroxyl radicals [25]. Peroxidation of membrane lipids alters the bi-layer thickness, membrane fluidity, and membrane permeability.
‑ ROS can directly damage deoxyribonucleic acids (DNA) by causing double strand breaks, structural changes, DNA mutations, or protein-DNA cross-links [26].
‑ They also regulate several apoptosis and necrosis signaling cascades. ROS can activate p53, a key molecule in ROS-induced cell death [27], which, in turn, upregulates PUMA (p53 upregulated modulator of apoptosis). ROS can open the mitochondrial permeability transition pore (MPTP), leading to mitochondrial swelling and cytochrome c release, thereby initiating apoptosis [28]. The MAPK (mitogen activated protein kinase) pathway, also triggered by ROS, has 3 main members: c-Jun NH2-terminal kinase (JNK), extracellular signal-regulated kinase 1/2 (ERK 1/2), and p38 MAPK. While ERK 1/2 has a controversial role in cell death and appears to be rather neuroprotective against ischemia/reperfusion injuries [15], JNK and p38 MAPK, activated by ROS through ASK1 (apoptosis signal-regulating kinase 1), significantly contribute to apoptosis during reperfusion after an ischemic insult [29,30].

### 2.1. Mitochondria as a Source of ROS and Their Implication in Cerebral Ischemia/Reperfusion Injuries

Mitochondria, the powerhouse of the cell, generate over 90% of the ATP in the brain through beta-oxidation of fatty acids, the Krebs cycle, and oxidative phosphorylation (OxPhos) [31]. They also use pyruvate from cytosolic glycolysis to reduce flavin adenine dinucleotide and nicotinamide adenine dinucleotide, which serves in energy transfer to the electron transport chain (ETC) [32].

The mitochondrial electron transport chain (ETC) consists of a series of protein complexes situated in the inner mitochondrial membrane which use the electrons removed by reduced nicotinamide adenine dinucleotide (NADH) and flavin adenine dinucleotide (FADH_2_) from the Krebs cycle to pump protons from the matrix into the intermembrane space, thereby generating a potential gradient across the inner mitochondrial membrane, which will be used in the final step of OxPhos to synthesize ATP [33]. NADH binds to NADH dehydrogenase (complex I), located on the inner mitochondrial membrane, and donates two electrons which will be passed down to ubiquinone to produce ubiquinol, a process coupled with the translocation of four protons from the matrix through the inner mitochondrial membrane [34]. Complex II, or succinate dehydrogenase, also participates in the Krebs cycle and contains FAD as a prosthetic group. It oxidizes succinate to fumarate and reduces ubiquinone [35]. Ubiquinol diffuses through the inner mitochondrial membrane and donates its electrons to cytochrome c reductase (complex III), which passes these electrons onto two molecules of cytochrome c while translocating two protons from the mitochondrial matrix and depositing an additional two protons in the intermembrane space [36]. At complex IV (cytochrome c oxidase), four cytochrome c molecules donate each one electron which will serve to form two H_2_O molecules from one O_2_ molecule, a process coupled with pumping of four protons from the matrix into the intermembrane space [37]. The final step is the synthesis of ATP from ADP and phosphate, achieved by ATP synthase (complex V), which uses the energy of the proton electrochemical gradient in a complex process, the elucidation of which led Boyer and Walker to achieve the 1997 Nobel Prize in Chemistry [38]. The transfer of protons from the mitochondrial matrix to the intermembrane space by the reactions of complexes I, III, and IV establishes a negative potential difference (ΔΨm) of 150–180 mV (with respect to the cytosol) across the inner mitochondrial membrane, which, together with the pH difference, drives complex V to generate ATP and cytosolic calcium ions to accumulate via the mitochondrial calcium uniporter in the matrix [32,39], where calcium stimulates the activity of dehydrogenases in the Krebs cycle and modulates the function of complexes IV and V [40]. As such, the balance between phosphorylation and dephosphorylation of the OxPhos complexes as well as intramitochondrial calcium concentrations maintain the cellular respiration rate and the ΔΨm [41] by interfering with their electron transfer kinetics and allosteric regulation by ATP and ADP (adenosine diphosphate) [42]. Under normal conditions, more than 90% of oxygen is reduced to water, while about 2% of electrons may leak from complexes I and III and react with oxygen, generating superoxide anions [41]. During ischemia, the intramitochondrial calcium levels increase [42], activating mitochondrial phosphatases and leading to dephosphorylation of the OxPhos complexes, especially of cytochrome c and of cytochrome c oxidase [43], and ultimately to loss of allosteric inhibition by ATP [41]. Because oxygen as the final electron acceptor is lacking, OxPhos is maximally activated in a feed-forward mechanism. Upon restoration of oxygen supply, increased OxPhos activity restores ΔΨm within 1 min and cellular levels of ATP within 15 min [44], after which it hyperpolarizes the mitochondrial membrane potential with dramatic effects on ROS production. Research has shown that a 10 mV increase in the ΔΨm above 140 mV leads to a 70–90% increase in the generation of ROS [45]. 

In ischemic conditions, the cellular energy deficiency leads to malfunction of the Na^+^/K^+^ ATPase pump and to neuronal membrane depolarization with additional release of excitatory neurotransmitters, especially glutamate [46]. This neurotransmitter binds mainly to two ionotropic receptors, which are ligand-gated ion channels: N-methyl-D-aspartate receptors (NMDARs), and α-amino-3-hydroxy-5-methylisoxazole-propionic acid receptors (AMPARs). In the resting state, the channel pores of NMDARs are blocked by magnesium and removed by the partial depolarization resulting from glutamate binding to AMPARs [47]. NMDARs are heterotetramers having two GluN1 subunits and two GluN2 subunits, which can be subclassified into GluN2A-GluN2D subunits [48]. Synaptic NMDARs are mainly GluN2A receptors, while the more recently identified extrasynaptic NMDARs are mainly GluN2B receptors [49]. Binding of glutamate to synaptic NMDARs promotes cell survival by activating phosphoinositide-3-kinase (PI3K), and phosphorylates Akt, which, in turn, phosphorylates and inactivates glycogen synthase kinase 3β (GSK3β), pro-apoptotic B cell lymphoma 2 (Bcl-2) associated death promotor Bad, apoptosis signal-regulating kinase 1 (ASK1), and apoptotic p53 [50,51]. By binding to extrasynaptic NMDARs, glutamate dephosphorylates and inactivates cAMP responsive element binding protein (CREB), inactivates the ERK pathway and promotes the expression of pro-death genes [52]. Glutamate binding to postsynaptic receptors promotes significant calcium influx, which cannot be expelled from the cells due to energy failure, and leads to increases in intracellular calcium concentrations. Increased cytosolic calcium activates Ca2+/calmodulin-dependent protein kinases (CAMKs), which phosphorylate NMDARs and AMPARs, further increasing the calcium influx [53]. 

Increased intracellular calcium concentrations also activate the ryanodine receptors and promote further calcium release from the endoplasmic reticulum [54]. Mitochondria attempt to buffer the cytosolic calcium levels, the ion being able to cross, along with other ions and metabolites, the outer mitochondrial membrane through the voltage-dependent anion channels (VDACs) and to further move into the mitochondrial matrix mediated by the mitochondrial calcium uniporter [33]. The excessive mitochondrial calcium concentrations additionally impair mitochondrial function by dissipating the ΔΨm, leading to an increase in ROS production and opening of the mitochondrial permeability transition pore (MPTP) [55].

The generated ROS damage mitochondrial components and lead to mitochondrial dysfunction. One important target of ROS appears to be cardiolipin, a dimeric phospholipid located in the inner mitochondrial membrane [56], where it interacts with complexes I, III, IV, V, and cytochrome c [57]. In the brain, cardiolipin is rich in unsaturated fatty acids such as arachidonic acid and docosahexaenoic acid [57], and, thus, prone to oxidative damage [58]. Cardiolipin peroxidation leads to its redistribution to the outer mitochondrial membrane [59], a 50% reduction in cytochrome c oxidase activity [60], and the release of mitochondrial apoptotic proteins such as cytochrome c, apoptosis inducing factor (AIF), Smac/DIABLO (second mitochondria-derived activator of caspase/direct IAP-binding protein with low PI) and the serine protease HtrA2/OMI (high-temperature requirement protein A2) into the cytosol [61]. The down-stream effects of these proteins will be discussed in the following sections.

Mitochondria contribute to cell death in the ischemic penumbra through many pathways. A series of pro-apoptotic proteins, such as Bid and Bax, belonging to the Bcl-2 family are upregulated during reperfusion [62]. Increased mitochondrial calcium cleaves Bid into truncated tBid, which interacts with other proapoptotic proteins at the mitochondrial membrane. Activated Bad translocates to the outer mitochondrial membrane and inhibits antiapoptotic proteins [63].

**Figure 1:**
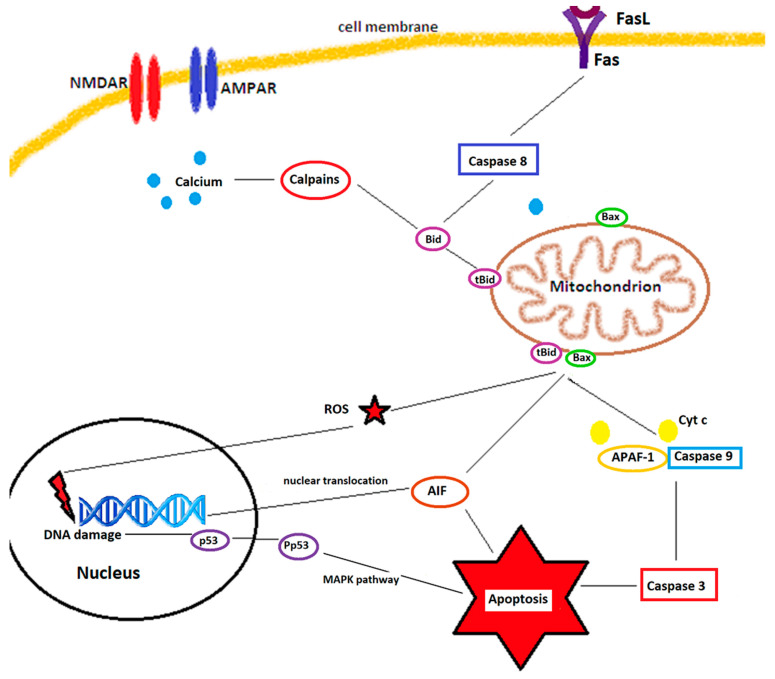
Mechanisms of ischemic neuronal cell death. Stimulation of the NMDA receptors (NMDARs) and AMPA receptors (AMPARs) increases the cytosolic calcium, which activates calpains and causes mitochondrial dysfunction. Binding of Fas ligands (FasL) to Fas death receptors activates caspase 8. Calpains and caspase 8 truncate Bid into tBid, which interacts with Bax at the mitochondrial membrane leading to pore formation and release of cytochrome c (Cyt c), apoptosis inducing factor (AIF), and reactive oxygen species (ROS). ROS and AIF, which translocate to the nucleus, mediate DNA damage and activate nuclear neuronal cell death pathways, such as phosphorylation of p53. Phosphorylated p53 (Pp53), via the MAPK pathway, leads to apoptosis. Cytochrome c, after being released from mitochondria, forms together with apoptotic protein activating factor-1 (APAF-1) and procaspase 9 the apoptosome. This will further activate executioner caspases, such as caspase 3, and lead to caspase-dependent apoptosis. Adapted from Culmsee and Krieglstein, [68].

Once the MPTP open, cytochrome c is released into the cytosol, where it binds to APAF-1 (apoptotic protease activating factor 1), and procaspase-9 and form the apoptosome, which ignites apoptosis. Two other proteins released from the mitochondrial intermembrane space, SMAC/DIABLO and Omi/HtrA2, promote caspase-independent apoptosis [64] by blocking the activity of IAP (inhibitor-of-apoptosis protein) family members, such as XIAP (X-linked inhibitor of apoptosis). Antiapoptotic proteins, such as Bcl-2 and Bcl-XL, inhibit the release of SMAC/DIABLO and subsequent inactivation of XIAP [65]. In experiments performed in rats, viral delivery of Bcl-2 blocks the nuclear translocation of AIF and improves cortical neuronal survival [66]. Caspase-8 and calpains, activated by the increased cytosolic calcium, cleave Bid (Bcl-2 interacting domain), which translocates to mitochondria and induces conformational changes in proapoptotic proteins such as Bax, Bad, or Bcl-XS, while inactivating antiapoptotic proteins, such as Bcl-2 or Bcl-XL [67]. Figure 1 shows schematically the multiple ways of cell death after ischemia.

Mitochondria are dynamic organelles, with number, size, and shape regulated by fission and fusion. Fission helps maintaining an adequate number of organelles by creating new mitochondria, but is also involved in removal of damaged mitochondria, while fusion is the process through which the membranes of two or more mitochondria merge and allow their content to mix, thereby sharing essential components and allowing for partially damaged mitochondria to be repaired. Fission manifests as constriction and cleavage of the mitochondria and is regulated by Drp-1 (dynamin-related protein 1), a cytosolic GTPase (guanosine triphosphatase) which is normally recruited to the outer mitochondrial membrane to initiate fission. During ischemia, ROS can phosphorylate and activate Drp-1 [69]. Following phosphorylation, Drp-1 moves to the outer mitochondrial membrane, interacts with other proteins such as mitochondrial dynamics proteins (MIFs), mitochondrial fission factor (MIFF), and human fission factor -1 (FIS-1) [70], and oligomerizes into a structure which constricts the mitochondria [63]. The mechanism is more complex, because the endoplasmic reticulum, by contacting the mitochondria, marks the site of constriction [71]. Under normal conditions, the process is essential for providing growing and dividing cells with adequate number of mitochondria, but fission is also involved in mitochondrial quality control and apoptosis [72]. Downregulation of Drp1 reduces the infarct size after focal cerebral ischemia [73]. Mitochondrial fusion is regulated by Opa1 (optic atrophy protein 1) for the fusion of the inner membrane and mitofusins Mfn1 and Mfn2 for the fusion of the outer membranes [74]. Under normal conditions, this process increases mitochondrial viability by sharing essential components [75]. Fusion is less extensively studied in cerebral ischemia, although in experiments performed in vitro hypoxia decreased Mfn2 expression [76]. 

Mitochondria have also a pivotal role in cellular recycling through autophagy, by delivering damaged organelles and proteins to lysosomes for final degradation through a multi-step process regulated by a series of signaling pathways such as the beclin-1/class III PI3K (phosphatidylinositol-3 kinase), AMPK (adenosine monophosphate-activated protein kinase)/mammalian target of rapamycin (mTOR), and PI3K/Akt/mTOR pathways [77]. In the initial phase of ischemia, activation of autophagy plays a protective role by preventing dysfunctional mitochondria from releasing cytotoxic substances [78], the process being further upregulated by reperfusion [79]. However, long-term upregulation of autophagy will lead to increased cell destruction [77].

In addition, mitochondrial metabolism, MPTP responsiveness, and calcium sensitivity varies with age and gender [80], which may explain the worse outcome of vascular events in the elderly.

### 2.2. Nitric Oxide Synthases as Sources of Reactive Oxygen Species

Nitric oxide (NO) is synthesized through a reaction catalyzed by nitric oxide synthases (NOS). Three types of nitric oxide synthases are expressed in the central nervous system [81]: neuronal NOS (nNOS, or type 1) and endothelial NOS (eNOS, or type 3), which are calcium-dependent and produce nanomolar levels of NO, and inducible NOS (iNOS, or type3), which is calcium-independent and produces micromolar levels of NO [81,82].

In the early stages of ischemia, the decrease of blood flow increases the activity of eNOS leading to the production of small amounts of NO, which regulate blood flow and protect the brain microvasculature. However, glutamate excitotoxicity and the subsequent cellular calcium overload trigger NO production by nNOS, which exerts neurotoxic effects. Following reperfusion, upregulated expression of iNOS via the NF-κB (nuclear factor- κB) pathway [83] leads to excessive amounts of NO, which can last for up to 7 days [84]. The dual neuroprotective and neurotoxic roles of NO have been convincingly demonstrated by genetic and pharmacologic approaches. Compared to wild-type mice, eNOS knockout mice exhibited larger infarct sizes [85]. Knockdown of the nNOS or iNOS gene resulted in a neuroprotective effect in mouse models of transient or global ischemia [86,87], as did pharmacologic inhibition of these enzyme isoforms [88,89]. 

NO is also involved in the breakdown of the blood–brain barrier (BBB) and brain edema formation. Schematically, the constituents of the BBB are the astrocytic end feet, the extracellular matrix, and the endothelial cells connected through tight junctions due to the presence of JAM-1 (junction adhesion molecule-1), claudins, and occludin, the cytoplasmic domains of which are anchored to the cytoskeleton through proteins of the zona occludens family [81]. Matrix metalloproteinases (MMPs) weaken the BBB by hydrolyzing tight junction and extracellular matrix proteins. MMP-2 is upregulated immediately after cerebral ischemia [90], whereas MMP-9 activity is increased in later stages [91]. Overexpression of eNOS or the pharmacological use of an NO donor has been shown to inhibit the expression of MMP-2 in endothelial cells [92]. 

In addition, by reacting with superoxide, NO leads to the formation of peroxynitrite (ONOO^−^), a highly reactive radical which can easily penetrate lipid membranes and exert neurotoxic effects through lipid membrane peroxidation, protein tyrosine nitration, activation of PARP (poly-ADP-ribose polymerase) and induction of mitochondrial dysfunction [93]. Moreover, uncoupling of eNOS from tetrahydrobiopterin, as occurs under ischemic conditions, leads to generation of superoxide instead of NO [94]. 

### 2.3. NADPH Oxidase as a Source of ROS

NADPH oxidase (NOX) is an enzymatic complex which transfers electrons from NADPH to oxygen molecules across the cell membrane, thereby generating superoxide [23]. The key step in activation of NOX is phosphorylation by protein kinase C isoforms, protein kinase B, p38, or the p21 mitogen activated kinases (MAPK) [95]. The vascular NOX isoforms have lower activities, producing ROS used mainly in signaling cascades, but following ischemia and especially after reperfusion they are able to produce high levels of ROS which increase oxidative stress [95]. It appears that glucose, not oxygen, increases the activity of neuronal NOX [96], which may explain the detrimental effect of hyperglycemia on stroke outcome or the worse prognosis of stroke in diabetic patients, who have activated NOXs.

### 2.4. Xanthine Oxidase as Source of ROS

Xanthine oxidoreductase catalyzes the oxidation of hypoxanthine to xanthine, and of xanthine to uric acid, while reducing NAD^+^ or molecular oxygen [97]. In the resting state, xanthine oxidoreductase exists as xanthine dehydrogenase, which prefers NAD^+^ as an electron donor. During reperfusion, oxidation and proteolysis converts xanthine dehydrogenase to xanthine oxidase, with higher affinity for oxygen as an electron donor, and which metabolizes hypoxanthine and xanthine, generating hydrogen peroxide [98]. 

## 3. Antioxidative Signaling Pathways

Cells are also endowed with antioxidative signaling pathways, which exhibit complicated interplays with the cell death signaling pathways. 

### 3.1. The Nrf2/ARE Signaling Pathway

The nuclear factor erythroid 2–related factor 2 (*Nrf2*) belongs to the alkaline leucine zipper family [99]. It is a transcription factor which upregulates antioxidant proteins such as manganese superoxide dismutase, heme oxygenase-1 (HO1), NADPH quinone reductase 1, catalase, GPX, peroxiredoxin 1, and heat shock proteins [100], and inhibits proinflammatory cytokines, matrix metalloproteinases, iNOS, cyclooxygenase-2, and the expression of cell adhesion molecules [101]. 

**Figure 2:**
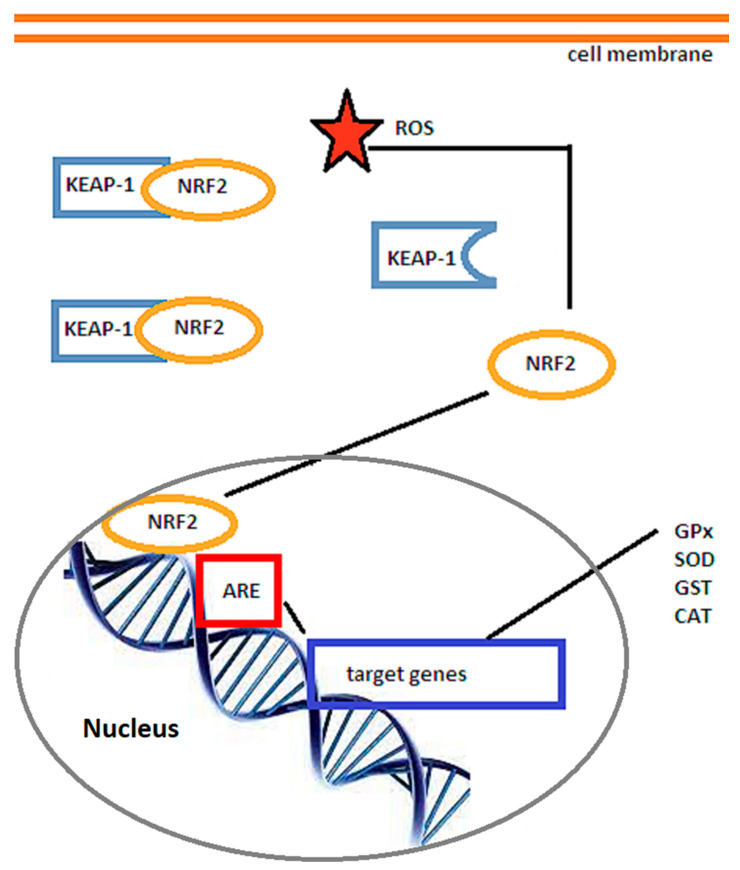
Schematic representation of the Nrf2/ARE signaling pathway. Under the influence of reactive oxygen species (ROS), the Nrf2/ARE complex dissociates and Nrf2 translocates to the nucleus, where it triggers the expression of genes with ARE (antioxidant response element) sequence in their promoters, leading to the production of endogenous antioxidants such as glutathione peroxidase (GPx), superoxide dismutase (SOD), glutathione S-transferase (GSH), and catalase (CAT). Adapted from Vasconcelos et al. [109].

In the quiescent state, Nrf2 is bound to Keap 1 (Kelch ECH associating protein 1) and inhibited. Subsequent to ROS attack or mediated through the proteinkinase C (PKC) pathway, MAPKs, or phosphoinositide 3-kinases (PI3Ks) [102], Keap 1 configuration changes and Nrf2 is uncoupled, being able to translocate to the nucleus, where it binds to ARE (antioxidant response element) and regulates the gene expression of antioxidant enzymes, growth factors, and anti-inflammatory proteins [103], as shown in Figure 2. The activation through the PI3K/Akt pathway occurs through inhibition of GSK-3β [104] and is triggered mainly by interleukin 4, which has been found significantly increased several hours after stroke [105]. In contrast, the p38-MAPK pathway can phosphorylate Nrf2 and strengthen its binding to Keap1 [106].

In vitro, Nrf2 exhibits neuroprotective properties, inhibiting glutamate toxicity and calcium overload [107,108]. In vivo, Nrf2-knockout mice are more susceptible to ischemic brain damage, brain edema, and have larger infarct volumes compared to wild type mice [100].

### 3.2. The SIRT/FOXO Signaling Pathway

Sirtuins (SIRT 1–7) are a group of evolutionary conserved NAD^+^ (nicotinamide adenine dinucleotide)-dependent lysine deacetylases and ADP ribosylases involved in many cellular processes, such as oxidative stress, apoptosis, or energy metabolism, and which are widely studied in ischemia/reperfusion injuries [110]. By sensing the NAD^+^ levels, sirtuins can initiate a series of adaptive responses, regulating the metabolic efficiency of the cell [111]. 

Glucose and oxygen deprivation during cerebral ischemia prevents cells from generating sufficient amounts of ATP, leading to failure of Na^+^/K^+^ ATPase, cellular depolarization, and glutamate release, which, by acting on NMDA and AMPA receptors, results in massive calcium and sodium influx. As shown above, increased intracellular calcium leads to increased ROS, which damage intracellular components, including nucleic acids. DNA damage is detected by poly-ADP ribose transferases (PARPs), which form poly-ADP ribose units from NAD^+^ to activate DNA repair. Overactivation of PARPs may consume up to 80% of intracellular NAD^+^ [111], limiting its availability for other enzymes, including sirtuins. 

SIRT 1 is located mainly in neuronal nuclei, as well as in astrocytes and microglia. It activates the FOXO family (class O forkhead box transcription factors) as well as the peroxisome proliferator-activated receptor γ coactivator 1. FOXO3a can increase the ROS scavenging activity [112]. In addition, SIRT 1 preserves blood flow and supports mitochondrial function. Its levels fall after cerebral ischemia and continue to decline following reperfusion [113]. Resveratrol, curcumin, or melatonin have been shown to increase SIRT 1 levels [111].

SIRT 6 is also a nuclear sirtuin, post-translationally modified by reactive nitrogen species. However, it appears that it has detrimental effects in cerebral ischemia, exacerbating necrotic cell death [111]. 

SIRT 3 is mainly a mitochondrial sirtuin, regulating energy metabolism and promoting neuroprotection against NMDA-mediated neurotoxicity through its downstream genes FOXO3a, SOD 2 (MnSOD), and catalase [114]. 

SIRT 2 is located mainly in the cytoplasm and deacetylates FOXO1 and FOXO3a, thereby exhibiting detrimental effects in cerebal ischemia. SIRT 4, 5, and 7 have not been consistently studied in cerebral ischemia. Research aimed at unraveling the actions of sirtuins after ischemic stroke yielded sometimes conflicting results, showing them to be involved both in neuroprotective pathways as well as in mechanisms which exacerbate tissue injury. Table 1 provides an overview of these various pathways.

However, it appears that the effects of SIRT 1 are NAD^+^-dependent [112]. Cerebral ischemia, by causing NAD^+^ depletion, could negatively impact SIRT 1 activity. The administration of nicotinamide, by preserving cellular NAD^+^ levels, can prevent neuronal excitotoxic death [113]. SIRT 3 enhances mitochondrial manganese superoxide dismutase (MnSOD) and catalase activity, both enzymes neutralizing ROS [114].

## 4. From Bench to Bedside: Translating Knowledge on Oxidative Stress in Ischemic Stroke into Therapeutic Approaches

Unfortunately, translating accumulated knowledge on the contribution of oxidative stress to cellular damage in ischemic stroke into more efficient therapies has proved a challenging endeavor. Despite success in preclinical trials, the clinical use of a series of molecules has failed or yielded inconclusive results, leading the scientific community to search for causes for these discrepancies in order to avoid future failures. Several factors have been identified [115], as discussed below.

Most preclinical studies were performed in animal models of transient ischemia, lasting for 1–2 h, and much less frequently in models of permanent cerebral ischemia. Only a small percentage of human patients achieve recanalization so early. The therapeutic time window for endovascular treatment was extended to 24 h [116], but many patients suffer reocclusions or recurrent stroke, and the success rate of recanalization depends on collateral flow as well [117].

The success rates in preclinical models are determined by infarct size, while in clinical trial the endpoint is functional outcome, and a significant discrepancy between infarct volume and clinical deficits may exist in human subjects [118]. 

Stroke usually occurs in elderly people, with significant comorbidities, while preclinical studies are performed mainly on young and healthy animals. Aging leads to a significant decline in the brain’s ability to restore its biochemical and cellular functions, resulting in a 9-fold increase in mortality rates in aged rats [119]. Human patients also exhibit higher mortality rates with increasing age [120], possibly related to higher levels of oxidative stress, mitochondrial dysfunction induced by aging, as well as the presence of comorbidities, such as diabetes or hypertension, which may influence the efficacy of neuroprotectant therapy [121]. In addition, patients can exhibit a series of post-stroke conditions, such as psychiatric disturbances, cognitive decline, and obstructive sleep apnea, which are not commonly assessed in animal models [122].

The strategies to pharmaceutically diminish oxidative stress following cerebral ischemia and reperfusion can be subdivided into the following sections.

### 4.1. Upregulation of Endogenous Antioxidant Defenses

Antioxidant vitamins C and E are among the most studied natural antioxidants. Observational studies in human patients showed that increases in plasma levels of vitamin C were correlated with reduced incidence of stroke [123]. In animal studies, although 4 weeks pre-treatment with vitamins C and E diminished the degree of lipid peroxidation and reduced infarct volume after middle cerebral artery occlusion [124], administering dehydroascorbic acid after arterial occlusion in baboons with transient cerebral artery occlusion was not able to significantly decrease lesion volume [125]. Similarly, human stroke trials with antioxidant vitamin supplements showed no benefits, neither in the acute setting [126] nor in prevention [127].

### 4.2. Preventing the Generation of ROS during Cerebral Ischemia

Another possibility would be to prevent the generation of ROS in ischemic tissue. From animal experiments it has become clear that excessive ROS production occurs during ischemia, followed by a second peak of ROS generation following reperfusion, suggesting an opportunity to interfere with this process [23].

1. NOX inhibition would be one possibility, although it is not yet clear which NOX isoform and which cell types play key roles in ischemia/reperfusion-induced ROS production [95]. NOX 2 promotes the production of superoxide, while NOX4 facilitates hydrogen peroxide production [128,129]. NOX2- and NOX4-deficient mice exhibited decreased infarct size compared to wild-type animals [130].

In animal experiments, a NOX inhibitor, VAS2870, showed favourable effects in terms of infarct size and neurological functions in mice [131], but subsequent studies showed that the compound had significant off-target effects and, by alkylating thiol moieties, may actually replicate some of the effects of ROS on cellular redox status [132]. Other molecules under investigation are gp91ds-tat [133] and GKT136901 [134]. The further development of selective NOX inhibitors, able to target specific isoforms and lacking off-target and side effects, could prove beneficial in human stroke.

2. Xanthine oxidase inhibitor allopurinol has long been used in humans to treat gout. It can also reduce superoxide formation [129]. Despite encouraging results in experimental studies [135], it did not show any benefit in clinical setting [136]. Other small molecules acting as XO inhibitors have been developed and are being tested, such as TEI-6720 [137], BOF-4272 [138], or febuxostat [139].
3. The generation of ROS by cyclooxygenases (COXs) and lipoxygenases (LOXs) have also been targeted in animal models of stroke. COX-2 knockout in mice and COX-2 inhibition with NS-398 resulted in reduced infarct size after middle cerebral artery occlusion [140,141]. Similarly, 12/15-LOX knockout or inhibition with baicalein or LOXBlock-1 led to reduced infarct size after transient middle cerebral artery occlusion in animal experiments [142,143].

### 4.3. Free Radical Scavengers

1. Lipoic acid (ALA, 1,2-dithiolane-3-pentanoic acid) recycles vitamin E and C [144] and is a free radical scavenger and a co-factor in the mitochondrial dehydrogenase complexes [145]. Pre-treatment with lipoic acid reduced infarct size [146] and improved functional recovery in animal models of stroke [147]. In a retrospective study of 172 thrombolysed acute ischemic stroke patients, 47 of which received alpha lipoic acid 600 mg/day, improved outcome was demonstrated both at 3 months and at one year [148]. Currently, another clinical trial, IMPORTANT, NCT 04041167, is recruiting diabetic acute ischemic stroke patients undergoing reperfusion therapies to assess the effect of the drug on outcome and complication rate [149].
2. NXY-059 (disodium 2,4-sulphophenyl-N-tert-butylnitrone, Cerovive), acts by adding a free radical to a nitrone spin trap, resulting in the generation of a spin adduct without the formation of free radicals, thereby terminating the radical chain reaction [129]. Despite reducing infarct size in a rat permanent middle cerebral artery occlusion model [150], improving functional outcome in monkeys [151], and reducing disability at 90 days when given within 6 h after stroke onset to 1722 patients in the SAINT I clinical trial [152], a subsequently conducted phase III clinical trial (SAINT II), which enrolled 3306 patients, failed to show clinical efficacy [153]. The discrepancy in the results was attributed to the poor BBB availability of NXY-059 [154] as well as to statistical weakness of the SAINT I trial [155].
3. Tirilazad (U-74006F) is a potent inhibitor of oxygen free radical-induced lipid peroxidation in microvascular and nervous tissue [156]. A meta-analysis of several experimental studies found that the molecule reduced infarct volume by almost a third and improved neurobehavioral score by almost 50% [157]. As such, the RANTTAS trial began enrolling 660 patients who were administered tirilazad 6 mg/kg/day for 3 days, started within 12 h from stroke onset, with disability measured at 90 days by the Glasgow Outcome Scale and Barthel Index set as primary outcome. The trial was prematurely terminated due to lack of efficacy [158]. A second trial, using higher doses (12–15 mg/kg/day) and given within 4 h from symptom onset [159], was stopped prematurely due to safety concerns raised by TESS II, a parallel study running in Europe. A meta-analysis of trials with tirilazad concluded that the drug actually increases death and disability after ischemic stroke [160].
4. N-acetyl cysteine is a glutathione precursor with a free thiol group, through which it can react with ROS [161]. In rat stroke models, it reduced infarct size and improved neurological score [162]. After reports of the beneficial effects of oral N-acetylcysteine in ischemic stroke [163], two phase 2 clinical trials (NCT 04918719 and NCT 04920448), with the drug administered intravenously, are planned but are not yet recruiting [149].
5. Citicoline is a natural compound which, by stabilizing cell membranes and preventing lipid peroxidation, acts as an antioxidant [164]. As with other antioxidants, it showed efficacy in animal models by reducing lesion volume [165]. However, the ICTUS phase 3 trial (NCT 00331890), the only citicoline trial listed with published results [149], failed to show efficacy in moderate-to-severe acute ischemic stroke [166].
6. Edaravone (5-methyl-2-phenyl-4H-pyrazol-3-one, or MCI-186) is a lipophilic free radical scavenger able to scavenge superoxide, hydroxyl, and peroxide radicals [167] and has been approved in Asia for the treatment of acute ischemic stroke since 2002 [23]. In experimental settings, it reduces MMP-9 activation and recombinant tissue plasminogen activator-induced blood–brain barrier damage in rodents [168], raising the possibility of extending the time window for thrombolysis. Indeed, in a clinical trial, edaravone improved the outcome of acute ischemic stroke patients if given simultaneously with reperfusion therapy, with 80% of patients showing “remarkable” or “good” recovery as compared to patients who received edaravone after alteplase [169]. A recent study which enrolled over 10,000 patients with acute ischemic stroke showed that edaravone given within 48 h after endovascular revascularization was associated with greater functional independence at hospital discharge, lower in-hospital mortality, and reduced intracranial bleeding after admission [170]. A clinical trial including edaravone administered in a cocktail, together with dexamethasone and argatroban, 30 to 60 min after thrombectomy (NCT 04202549) is ongoing, while another trial, NCT04817527, or EXISTENT, will explore the safety and efficacy of edaravone dexborneol for patients with acute ischemic stroke receiving endovascular therapy in extended time windows, but is currently not recruiting patients [149].
7. Melatonin is an endogenous molecule synthesized in the pineal gland. It has been shown to be able to efficiently scavenge oxygen-centered free radicals, inhibit oxidative damage of biological molecules [171], and upregulate antioxidant defenses such as glutathione reductase, glutathione peroxidase, catalase, and SOD [172]. The efficacy of 14 mg melatonin daily supplementation in acute ischemic stroke was planned to be evaluated in a phase 4 clinical trial (NCT 01863277), but the current status of the study is unknown [149].

### 4.4. Degradation of Free Radicals

1. Ebselen reacts with peroxynitrite radicals and inhibits glutathione peroxidase-like activity [129]. In rodent stroke models, Ebselen reduced lesion size and improved recovery [173], although if administered after onset of ischemia, the protective effect was more modest [174]. In a clinical setting, 302 patients who received Ebselen within 48 h from stroke onset and continued for 2 weeks showed a slightly better outcome at 1 month, but the difference between the active and placebo arm at 3 months failed to reach statistical significance [175].
2. Lubeluzole inhibits the glutamate-mediated nitric oxide synthase pathway, thereby reducing NO levels and peroxynitrite production [176]. In animal models, lubeluzole was able to reduce infarct size by 50% when given 15 min, and by one third when given 30 min after ischemia onset [177]. However, in clinical trials Lubeluzole failed to improve outcome and caused heart conduction disorders and Q-T prolongation [178,179].

### 4.5. Mitochondria-Targeted Antioxidants

Mitochondria, as the main generator of ROS in cerebral ischemia and reperfusion injuries, would be an appealing target. However, achieving high antioxidant concentrations in intracellular locations may prove a difficult task [180] and involves the conjugation of the antioxidant molecule with a lipophilic cation to promote diffusion through the mitochondrial membrane [129].

1. Coenzyme Q10, an endogenous antioxidant, failed in slowing down the progression of Parkinson’s disease, a neurodegenerative disease with significant involvement of oxidative stress in its pathophysiology [181,182].
2. Another mitochondria-targeted antioxidant is Mito-Q10, which is able to mildly uncouple mitochondrial respiration and phosphorylation and, thereby, reduce the mitochondrial generation of ROS as well as protect mitochondria from oxidative damage caused by hydrogen peroxide [183]. Although intensely investigated in cardiovascular disease and neurodegenerative diseases, it has not been evaluated in ischemic stroke.
3. Preventing MPTP opening with cyclophilin D was the subject of intense research in the 1990s for attenuating ischemia/reperfusion injuries in the liver, heart, and brain, but the risks often outweigh the benefits [184]. However, more recently, cyclosporin A analogues, such as alisporivir/debio-025, sanglifehrin A, or NIM811, are being tested in animal models for attenuated ischemia/reperfusion injuries in myocardium, liver, or brain [185].

### 4.6. Novel Experimental Approaches

1. The inhalation of gases is appealing because gases are able to rapidly penetrate biological membranes and diffuse into the cytosol, mitochondria, and even the nucleus [186]. Beneficial effects in terms of infarct size were obtained with hydrogen gas in transient middle cerebral artery occlusion [186], normobaric oxygen [187], or inhalation of nitric oxide [188].
2. SOD catalyses the conversion of superoxide to hydrogen peroxide, which is eliminated by catalase and glutathione peroxidase [189]. Overexpression of Cu/Zn SOD in transgenic mice and rats, or induced by gene therapy in mice, resulted in reduced apoptosis in transient focal ischemia models [190,191].
3. Nrf2-activating drugs, such as omaveloxolone or auranofin, are currently being evaluated. Nrf2 is one of the most important transcription factors, with pleiotropic actions [192] and would thereby induce endogenous cytoprotective pathways. In humans, omaveloxolone increased its plasma concentration 1 h after administration without significant side effects except for upper respiratory tract infections, nasopharyngitis, diarrhoea, nausea, and fatigue [193]. Auranofin is an oral anti-arthritic drug with Nrf2-dependent antioxidative action [194]. Sulforaphane, present in cruciferous vegetables such as broccoli, is also a potent Nrf2 stimulator, although these multi-targeted dietary antioxidants have a diminished bioavailability [195]. Research has revealed that Nrf2 activity peaked at 8 h after transient middle cerebral artery occlusion in the peri-infarct zone [196], followed by expression of down-stream antioxidants such as glutathione, thioredoxin, or heme oxygenase 1, which increased 24–72 h after reperfusion [196]. Brain edema and neuronal death were significantly decreased by Nrf2-inducing drugs in mouse and rat stroke models [100].
4. Upregulation of SIRT1 has also been shown to be neuroprotective against cerebral ischemia/reperfusion injuries [197]. Resveratrol was shown to upregulate the Sirt1-PGC-1α signaling pathways [198] as well as the expression of brain-derived neurotrophic factor (BDNF) [199]. However, it also has a limited ability to cross the BBB [195]. Similarly, SIRT6 overexpression in the brain of mice through gene transfer was able to reduce ischemia/reperfusion tissue damage, oxidative stress, and neurological deficits through increases of total and nuclear Nrf2 levels [108].
5. Leptin is a hormone previously studied in relation to energy expenditure and satiety. However, more recently, complex signaling cascades ignited by leptin binding to its receptors have been documented, including the Janus kinase (JAK)/signal transducer and activator of transcription (STAT), Ras/extracellular signal-regulated kinase (ERK)1/2, phosphoinositide-3 kinase (PI3K)/Akt/forkhead box O1, adenosine monophosphate kinase (AMPK) and mTOR/ribosomal protein 6 kinase pathways, through which leptin inhibits the release of presynaptic glutamate (JAK2/PI3K pathway) [200], promotes mitochondrial biogenesis (JAK/STAT pathway) [201], increases SOD levels [202] and has also an anti-inflammatory action (JAK2/STAT3 and p38MAPK/ERK1/2 pathways) [203]. Barriers imposed by drug pharmacokinetics [204] could be overcome by special formulations or synthetic polymers, delivered mainly to the damaged areas.
6. MicroRNAs (miRNAs) are non-coding RNAs which modulate gene expression at the post-transcriptional level [205]. Research has shown that in experimental middle cerebral artery occlusion in mice, miR-98-5p was downregulated in brain tissue samples, and that upregulation of miR-98-5p enhanced SOD activity and protected against oxidative stress through upregulation of the NAD(P)H quinone oxidoreductase 1 and heme oxygenase 1 levels [206]. In addition, miR-98-5p inhibited apoptosis by increasing Bcl-2 levels and by reducing Bax and cleaved caspase-3 levels [206]. Other microRNAs, such as miR133b [207] or miR-124-3p [208], can modulate brain plasticity and neuroinflammation following ischemic stroke [209], contributing to angiogenesis, neurogenesis, oligodendrogenesis, and astrogenesis, and improve functional recovery if administered in a prolonged time window after the ischemic insult (days to months after stroke) [210]. Technical issues relating to the appropriate timing of mesenchymal stem cell transfer, the route of administration, and the characterization of the stem cells need to be solved [211], but clinical trials have already been carried out on a small scale and show the therapy to be safe and to lead to significant functional improvement [212]. Similar effects were obtained with mesenchymal stem cell-derived extracellular vesicles, which are easier to deliver and contain proteins, lipids, nucleic acids with various forms of RNA, and membrane receptors. They are less immunogenic than stem cells and have lower risk of microvascular thrombosis, are able to cross the blood–brain barrier, and the miRNAs contained can be easily genetically modified [209].
7. Novel drug design, by dispersing or dissolving the drug within a polymer matrix, entrapping the drug inside lipid vesicles, encapsulating or adsorbing the active molecules on the surface of nanoparticles, are able to improve drug pharmacokinetics, pharmacodynamics and safety, and prevent off-target interactions [213]. Their small size, stability, long serum half-life, and ability to cross the BBB makes them a promising approach to deliver antioxidants in acute ischemic stroke [214], especially since in this setting activation of immune cells and the compromised BBB integrity potentiates the leakage of molecules [215]. The biological activities of nanomaterials, 1–500 nm in size, are related to their size, shape, and surface modifications [216]. They can be subdivided into [215]:
‑ Inorganic nanoparticles, such as cerium oxide nanoparticles, platinum nanoparticles, gold nanoparticles, or superparamagnetic iron oxide nanoparticles;
‑ Carbon allotropes, either spherical (fullerenes) or carbon nanotubes;
‑ Lipid-based nanoparticles, such as liposomes, non-liposomal lipid nanoparticles (polyethylene glycol, i.e., PEGylated lipid nanoparticles, nanostructured lipid carriers, and nanoemulsions);
‑ Polymer-based carriers, including micelles, dendrimers (branched polymers surrounding in repeated layers a central molecule), and nanogels.

**Figure 3:**
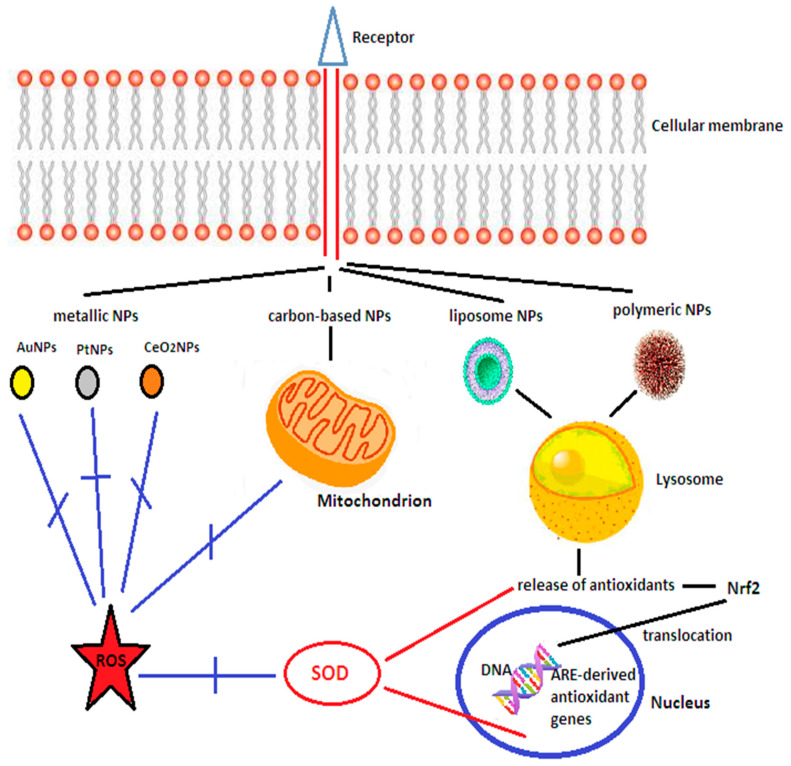
Schematic presentation of the action of the main categories of nanomaterials. Nanoparticles enter the cell through receptor-mediated endocytosis. Metallic nanoparticles (AuNPs, PtNPs, and cerium oxide NPs or nanoceria) have free radical scavenging properties. PtNPs also mimic SOD and CAT activity. Nanoceria downregulate iNOS and reduce the amount of nitric oxide and peroxynitrite. Carbon-based nanomaterials have SOD-like activity and protect the mitochondria from free radical damage. Liposome and polymeric nanoparticles can carry antioxidant molecules, enzymes, and genes which can activate Nrf2 and promote its nuclear translocation, where it binds to antioxidant response element (ARE) leading to the expression of ARE-derived antioxidant genes. NPs—nanoparticles; AuNPs—gold nanoparticles; PtNPs—platinum nanoparticles; CeO₂NPs—nanoceria; Nrf2—nuclear factor-erythroid 2-related factor 2; SOD—superoxide dismutase; DNA—deoxyribonucleic acid; ROS—reactive oxygen species; ARE—antioxidant response element; Blue lines indicate inhibitory activity, while red lines indicate activation. Adapted from Song et al. [216].

PEGylated cerium nanoparticles (NPs) and platinum NPs have been shown to have ROS scavenging properties [215,216], while superparamagnetic iron oxide NPs have been used to deliver under magnetic field guidance endothelial progenitor cells to potentiate angiogenesis [217]. Fullerenes have free radical scavenging potential, while carbon nanotubes were able to carry neural progenitor cells to the lesion site, which differentiated and were integrated with the surviving cells promoting improved behavioral outcomes [215]. Liposomes have been designed to carry dexamethasone, t-PA, or lycopene to diminish inflammation and oxidative stress [215], while studies with micelles loaded with SOD, catalase, curcumin, luteolin, quercetin, resveratrol, dexamethasone, nerve growth factor, or the nitric oxide radical amino-TEMPO also showed reduced levels of oxidative injuries, decreased neurological deficits and diminished BBB disruption [195,215]. Dendrimers are more often used to target the neuroinflammatory pathways [218]. Table 2 provides an overview of the studies performed and results obtained with nanoparticles in vitro and in animal models of stroke and Figure 3 illustrates schematically the action of the various nanomaterials.

The encouraging results of carbon nanoparticles or SOD nanoparticles in animal models of stroke open new therapeutic opportunities [237] and, possibly, reconsideration of previously failed antioxidant molecules in clinical trials.

## 5. Concluding Remarks

Given the complex interplay between the various cascades which contribute to tissue damage following cerebral ischemia and reperfusion, and the step-wise progression of these vicious pathways extending over days to weeks after the ischemic insult, it is likely that the one drug/one target approach will not yield rewarding results. Multi-targeted drugs, activation of the endogenous protective mechanisms, and a sequential approach with combination of multiple molecules seem more promising. A meta-analysis of 126 trials of treatments in animal stroke models showed that in terms of infarct size, single treatments reduced it by 20% and adding a second treatment offered an additional 18% reduction, while in terms of functional outcome, single treatments improved it by 12% with a further 25% improvement achieved by adding a second treatment [238,239].

In our view, antioxidants will have a definite place in the future of ischemic stroke treatment. Multimodal molecules, such as polyphenols, quercetin, or curcumin, with improved delivery (engineered as nanoparticles) and delivered prior to and together with recanalization therapies will allow antioxidants to diminish the magnitude of reperfusion injuries. Autologous stem cells could be harvested upon arrival of the patient in the stroke unit and cultured to allow transferring of healthy mitochondria to damaged cells [240], to be used in the subacute/chronic phase following stroke to modulate post-stroke neuroinflammation [241] and enhance neuro- and synaptogenesis, thereby improving functional recovery.

## Figures and Tables

**Table 1:** Pathways of neuroprotection and neuronal injury mediated by sirtuins.

Pathways of Cerebral Tissue Protection Following Ischemia	Sirtuins Involved
Reduces oxidative stress	SIRT 1, 3, 6
Promotes glutamate uptake	SIRT 4
Increased calcium buffering	SIRT 3
Preserves mitochondrial respiration	SIRT 1, 3, 5
Maintains ATP levels	SIRT 3, 4
Promotes neural stem cell proliferation	SIRT 1, 2
Promotes neural stem cell differentiation	SIRT 1, 2, 6
Preserves blood flow	SIRT 1
Maintains blood brain barrier integrity	SIRT 3
DNA repair	SIRT 1
Reduces inflammation	SIRT 1
Mediates neuroprotective effects of NAD^+^	SIRT 1, 5
**Pathways of Cerebral Injury Following Ischemia**	**Sirtuins Involved**
Increases oxidative stress	SIRT 2, 3, 6
Damages mitochondrial respiration	SIRT 3
Increases autophagy	SIRT 6
Induces axonal injury	SIRT 2

**Table 2:** Nanomaterials used to target ischemic stroke-induced oxidative stress. Adapted from Song et al. [216] and Dong et al. [219].

Nanoparticles	Drugs	Therapeutic Effect	References
PtNPs	-	Reduces superoxide generation and MMP9 activation, diminishes infarct volume	[220]
Nanoceria	- or Edaravone	Diminishes oxidative stress, scavenges ROS, reduces neuronal apoptosis, downregulates iNOS	[216,221,222,223]
Carbon nanotubes	Caspase-3 siRNA	Gene silencing of caspase-3, improved neuronal survival	[224]
Liposomes	Dexamethasone + tPA	Reduces infarct volume	[225]
	lycopene	Inhibits NOX2, reduces the levels of NO	[226]
	Vitamin C, E	Reduces oxidative stress	[227]
Polymer nanoparticles	edaravone	Free radical scavenging	[228]
	SOD	Reduces oxidative stress, diminishes infarct size	[229]
	curcumin	Reduces oxidative injury	[230]
	resveratrol	Diminishes lipid peroxidation	[231]
	N-acetylcysteine	Increases antioxidant activity	[232]
	Amino-TEMPO	Reduces oxidative injury and BBB disruption	[233]
	Quercetin	Reduces mitochondrial damage	[234]
	Heme oxygenase- 1 +/− dexamethasone	Reduces oxidative stress, diminishes ischemic brain damage	[235,236]

Abbreviations: NPs—nanoparticles;; Pt—platinum; SOD—superoxide dismutase; NOX—NADPH oxidase; NO—nitric oxide; iNOS—inducible nitric oxide synthase; ROS—reactive oxygen species; tPA—recombinant tissue plasminogen activator; MMP9—matrix metalloproteinase 9; siRNA—silencing RNA.
